# Myoid gonadal stromal tumor of the testis—the novel subtype of testicular gonadal stromal tumors: a case report and review of the literature

**DOI:** 10.1186/s13256-024-04393-7

**Published:** 2024-02-22

**Authors:** Klaus-Peter Dieckmann, Lars Tharun, Markus Angerer, Alexander Harms, Christian Wülfing

**Affiliations:** 1 Testicular Cancer Unit, Department of Urology, Asklepios Klinik Altona, Paul Ehrlich Strasse 1, 22763 Hamburg, Germany; 2 Medizinisches Versorgungszentrum Hanse Histologikum, Fangdieckstrasse 75a, 22547 Hamburg, Germany

**Keywords:** Testicular neoplasm, Gonadal stromal tumor, Testis-sparing surgery, Desmin, S100 protein, Smooth muscle actin, Inhibin

## Abstract

**Background:**

Sex cord gonadal stromal tumors compose less than 10% of all testicular neoplasms and consist of a variety of histological subtypes. In 2016, the World Health Organization introduced a novel subtype, the myoid gonadal stromal tumor, that consists of spindle-shaped cells with immunohistologic features of muscle cells. Only few cases have been reported to date. Due to its rarity and owing to its only recent introduction, the current knowledge about myoid gonadal stromal tumor is limited, and particularly, appropriate clinical management is still ill-defined.

**Case presentation:**

A 47-year-old man of Caucasian descent presented with nonspecific scrotal discomfort. A roundish and well demarcated hypoechoic mass of 8.5 mm in diameter was detected in the cranial region of the left testis. Serum tumor marker levels were within normal ranges. Testis-sparing surgery revealed a 9-mm whitish, hard mass with sharp surgical margin. Histologically, the neoplasm consisted of microfibrillar tissue with spindle-shaped cells harboring elongated nuclei. Immunohistochemical work-up disclosed expression of desmin, small muscle actin, and S100 protein giving evidence for the myogenic nature of the neoplastic cells. There was no indication of malignancy, neither histologically nor clinically. Follow-up of 1 year was uneventful.

**Conclusion:**

A literature survey revealed 22 previous cases of myoid gonadal stromal tumor. The median age was 37 years, the median size of the neoplasm was 20 mm, and there was no side-preponderance. Myoid gonadal stromal tumor is not much different from other subtypes of gonadal stromal tumors nor from testicular gem cell tumors regarding age and laterality; however, tumor size is smaller in myoid gonadal stromal tumors than in germ cell tumors. Although rarely performed so far, testis-sparing surgery probably constitutes an appropriate treatment of this neoplasm. Myoid gonadal stromal tumor represents an emerging novel entity of benign testicular new growths that caregivers of patients with testicular tumors should be aware of.

## Introduction

Testicular tumor is a rare disease with no more than 4100 cases arising annually in Germany [1]. Histologically, around 90% of testicular neoplasms comprise of germ cell tumors (GCTs), while the remainder is composed of a variety of rare neoplasms involving spermatocytic tumors, hematological neoplasms, sex cord gonadal stromal tumors, secondary malignancies, and others [2]. Sex cord gonadal stromal tumors comprise around 5–6% of all testicular neoplasms, most of which are of benign nature. Pathogenetically, these tumors are derived from tissue that originally formed the genital ridge during embryogenesis, which developed into the gonad by building the framework and seeding soil for the primordial germ cells invading from the yolk sac during the 4th to 6th week of gestation [3]. The most common neoplasm of this subgroup is the Leydig cell tumor, followed by several variants of Sertoli cell tumors [4, 5], Granulosa cell tumors [6, 7], mixed forms, tumors not otherwise specified (NOS), and extremely rare theca cell tumors [8]. Myoid gonadal stromal tumor (MGST) represents a newly recognized subtype among the sex cord gonadal stromal tumors. It is composed of cells sharing features of smooth muscle and of gonadal stroma as stated by the World Health Organization (WHO) expert team for classification of testicular tumors in 2016 [9]. The neoplasm is considered to derive from peritubular myofilaments or intertubular primitive mesenchymal cells. It is morphologically distinct from other sex cord gonadal stromal tumors [10], and it is characterized by spindle-shaped pale cells that are closely connected and surrounded by collagen layers [11]. Entrapping of seminiferous tubules by the neoplasm is considered to support the origin from peritubular structures. Immunohistochemical hallmarks are the expression of S100 protein, smooth muscle actin (SMA), and desmin.

Secondary to both its rarity and only very recent recognition as its own entity, the clinical features of MGST are still poorly understood, and no clear recommendations for treatment are available. Here, we report another case of MGST. In addition, a literature survey was performed to look for previously reported cases by searching the electronic data bases PubMed and Google Scholar and by hand-searching reference lists of previous reports. We discuss the clinical features of MGST in comparison with other testicular tumor entities.

## Case presentation

**Fig. 1:**
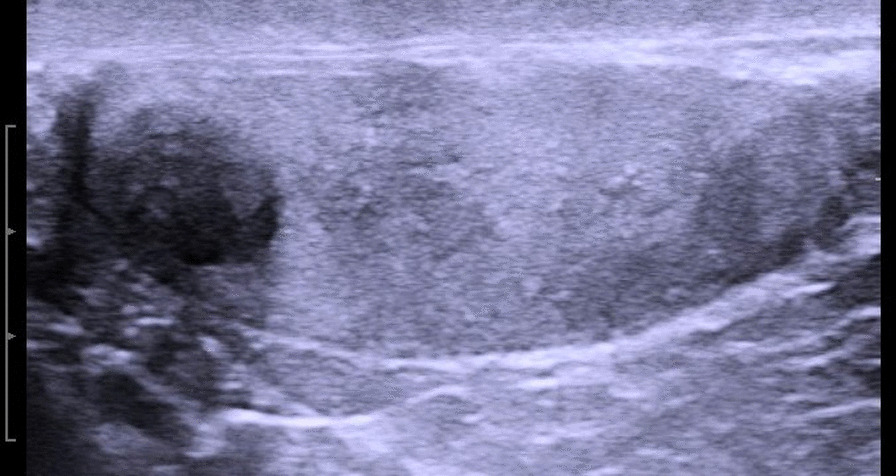
Sonographic appearance of myoid gonadal stromal tumors. Note the homogeneous well-demarcated hypoechoic mass at the cranial pole of the testicle

A patient of Caucasian descent, aged 47 years, presented with unspecific left-sided scrotal discomfort lasting for several weeks. The right testicle had been excised for unknown reasons, presumably for undescended testis with congenital hernia, in early childhood. The patient was unmarried and childless. He reported a sedentary lifestyle due to his profession as a software-developer. Physical examination did not disclose any major pathological finding except for obesity (body mass index 33.1 kg/m^2^). The right sided scrotal contents was empty. The left testicle was normal sized but very touch sensitive; thus, a proper palpation was not feasible. Sonographically, a well demarcated hypoechoic roundish mass of 8.5 mm in diameter was detected (Fig. 1). Color-coded Duplex sonographic signals were present within the mass. Laboratory workup showed serum tumor markers alpha fetoprotein, beta human chorionic gonadotropin, and lactate dehydrogenase within normal limits; the same applied to white blood count, hemoglobin, retention parameters, and liver enzymes. Serum testosterone level was subnormal with 1.75 ng/ml [reference limits (RL) 2.4–7.8 ng/ml] at normal luteinizing hormone level [6.80 IU/l (RL 1.14–8.75 IU/l)]. Spermatogenetic parameters revealed seriously impaired fertility with a follicle stimulating hormone level of 18.71 IU/l (RL 0.95–11.95 IU/l) and an inhibin B of 10 ng/l (RL 120–400 ng/l). Based on the finding of a well-vascularized intratesticular mass upon ultrasonography, a testicular tumor was suspected. Accordingly, inguinal surgical exposure was performed with cord clamping and frozen-section-guided testis sparing excision (TSS) of the mass along with two marginal testicular biopsies, as recently advocated in a major review article [12].

**Fig. 2:**
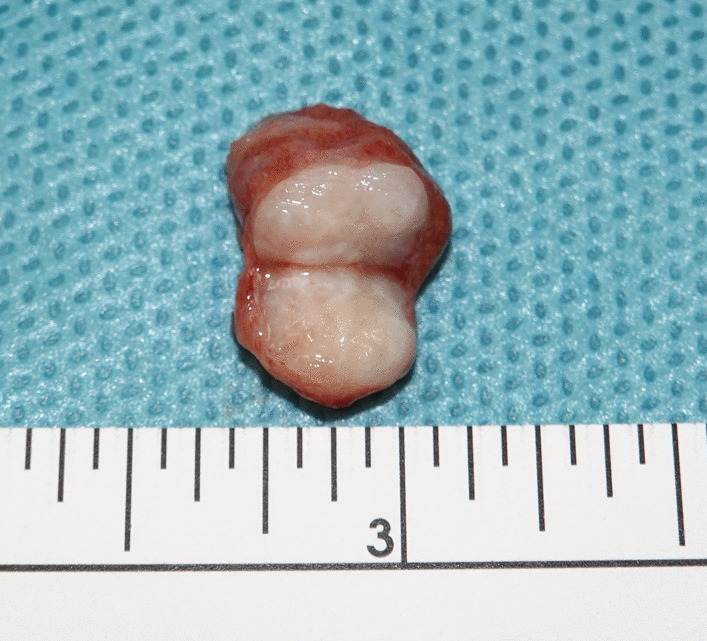
Surgical specimen: excisional biopsy from the testis. Note the whitish homogeneous mass with well-demarcated margins

**Fig. 3:**
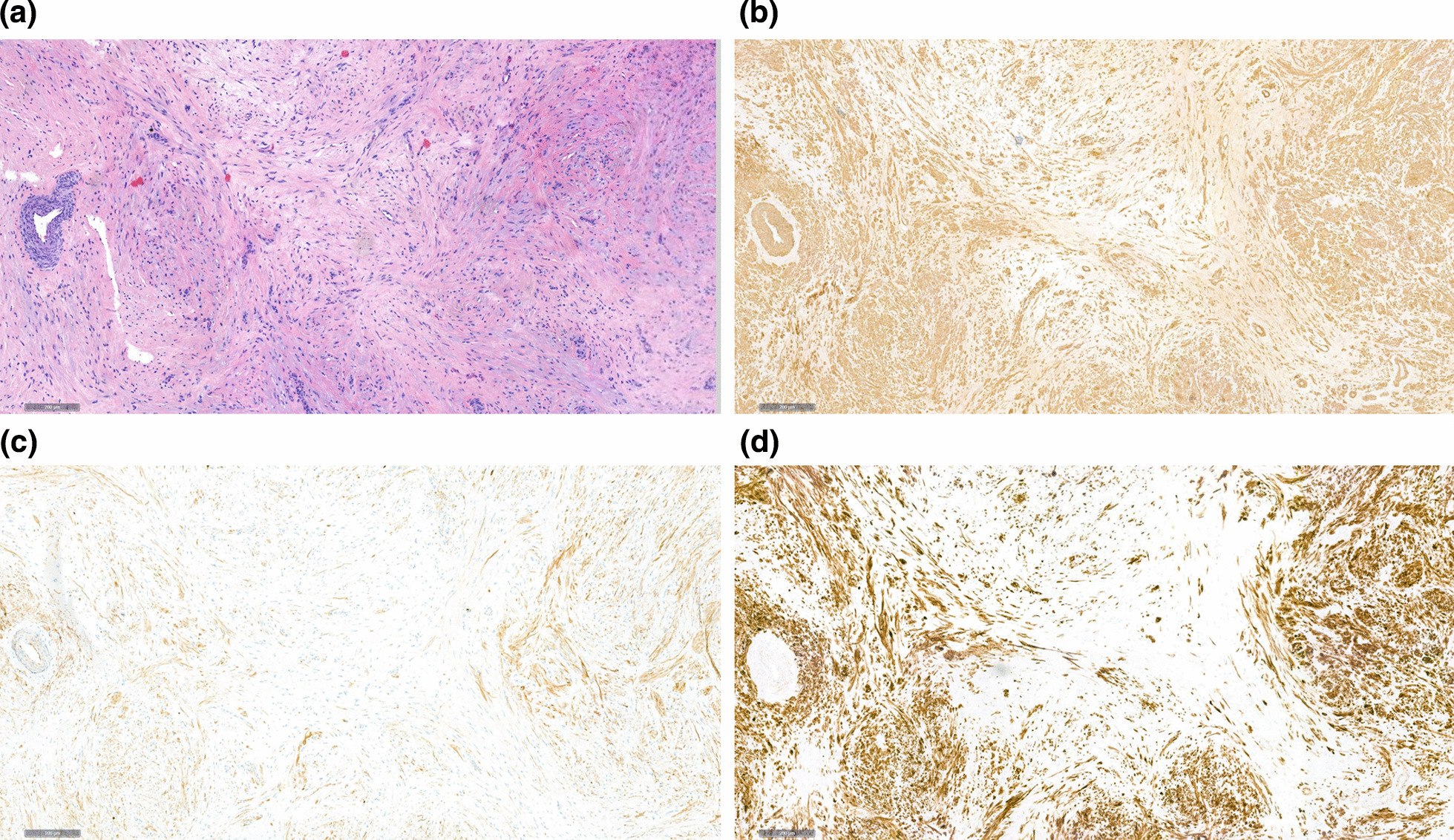
**a** The tumor consists of fascicularly oriented spindle cells, which exhibit moderate eosinophilic cytoplasm and inconspicuous nuclei (hematoxylin–eosin; ×40 magnification). **b** Immunohistologic staining: the neoplastic cells are consistently positive for SMA (smooth muscle actin; ×40 magnification). **c** Immunohistologic staining: evidence of S100 expression (S100; magnification ×40). **d** Immunohistologic staining: strong expression of desmin (desmin; magnification ×40)

On gross pathologic examination, the excised nodule consisted of a hard homogeneous mass of white-greyish color, sized 9 mm, with sharply demarcated surgical margin (Fig. 2). The adjacent testicular parenchyma was of normal brownish color and normal granular structure. Upon histological examination, the mass was homogeneously composed of microfibrillar tissue consisting of spindle-shaped cells with elongated nuclei (Fig. 3a). Immunohistologic examination revealed expression of desmin, SMA, and S100 but no expression of SOX10, inhibin, and calretinin (Fig. 3a–d). KI-67 proliferation index was < 1%. The marginal biopsies revealed atrophic testicular tissue with arrested spermatogenesis and focal Leydig cell hyperplasia but no germ cell neoplasia *in situ*. The final diagnosis was myoid gonadal stromal tumor.

The postoperative course was uneventful. Since no signs of malignancy had been found histologically and because computed tomography of chest and abdomen had not disclosed any abnormalities, no further treatment was administered. The patient was well and without local and distant recurrence 1 year after surgery.

## Discussion

Following the formal recognition of myoid gonadal stromal tumors as its own entity by the WHO in 2016 [9], several cases published earlier in literature were retrospectively identified as neoplasms fulfilling the definition of MGST. The first case description is credited to Evans in 1977 [13]. The term myoid gonadal stromal stromal tumor was coined by Weidner in 1991 [14]. Additionally, another two cases with peritubular myofilament expression were documented by Greco *et al.* in 1984 [15].

To the best of our knowledge, a few more than 20 cases of myoid gonadal stromal tumors have been reported in literature to date. As stated earlier, a number of cases documented earlier would probably match the morphological definition of MGST outlined by the WHO in 2016 [9, 16], but in the absence of unequivocal pathohistological guidelines at the time of reporting and due to the lack of immunohistological techniques, these early cases will stay buried in literature. The cases reported thus far, along with their clinical features, are listed in Table 1 [10, 11, 13–26]. The median age of reported cases of MGST is 37 years with an interquartile range of 24 to 45 years. The median tumor size is 20 mm, with an interquartile range of 13–30 mm. There appears to be no side preponderance; because of 16 cases with information on laterality, 8 were right sided and 8 left sided. With respect to surgical treatment, all but one had full orchiectomy, while one had TSS; one case was without information.

**Table 1:** Synopsis of previously published cases of myoid gonadal stromal tumors

First author	Year	Ref. (no.)	Age (years)	Tumor size (mm)	Side l/r	Treatment	Outcome
Evans	1977	[11]	4	35	L	ox	3 m NED
Greco	1984	[13]	24	20	NA	ox	12 m NED
			48	35	NA	ox	32 m NED
Miettinen	1986	[15]	52	37	NA	ox	3 m NED
Allen	1990	[16]	34	NA	NA	ox	36 m NED
Weidner	1991	[12]	46	25	R	ox	12 m NED
Nistal	1996	[17]	16	13	L	ox	NA
Renshaw	1997	[18]	45	21	NA	ox	60 m NED
Magro	2007	[19]	23	20	R	ox	13 m NED
Du	2012	[20]	25	15	R	ox	12 m NED
Kao	2014	[9]	38	12	L	ox	5 m NED
			43	13	R	ox	31 m NED
			59	32	R	ox	58 m NED
Renne	2021	[10]	53	40	R	ox	70 m NED
			31	20	R	ox	34 m NED
			41	10	L	ox	12 m NED
			37	10	L	ox	2 m NED
Ercolino	2022	[21]	20	6	R	TSS	12 m NED
D´Abbronzo	2022	[22]	41	10	L	NA	36 m NED
Madendere	2023	[23]	24	25	L	ox	NA
Elousrouti	2023	[24]	27	30	L	ox	NA
present case	2023		47	9	L	TSS	12 m NED

__________________________________________________________________________________

*ref.* reference, *L* left side, *R* right side, *NA* not available, *m* months, *NED* no evidence of disease, *ox* orchiectomy, *TSS* testis-sparing surgery

With 47 years of age, our case belongs to one of the oldest patients with MGST, since only four of the hitherto reported cases were older. With respect to tumor size, the present case is one of the smallest. Only one of the reported cases had a smaller size (6 mm) [23]. Thus, the clinical features of our case are different from average characteristics of MGST but still within reported ranges. With respect to treatment, our patient underwent TSS. This conservative surgical approach had been performed in only one previous case [23]. In our case, the main reason for TSS was the tumor location in a solitary testis.

Table 2 provides a comparison of the clinical features of MGST as abstracted from Table 1 with those of three other types of gonadal stromal tumors [5, 6, 27] and of testicular GCTs [28, 29].

**Table 2:** Clinical features of myoid gonadal stromal tumors—comparison with other testicular tumor entities

Testicular tumor entity	Data abstracted from	(*n*)	Age, median (years)	IQR	tumor size, median (mm)	IQR	Laterality right sided	TSS
Myoidal gonadal stromal tumor	Present report	23	37	24; 45	20	11; 31	50%	10%
Leydig cell tumors	Ruf 2020 [25]	208	41	33; 48	12.5	7; 20	51.5%	56%
Sertoli cell tumor	Grogg 2020^a^ [4]	435	29	16; 44	20	12.5; 39	NA	13%
Adult granulosa cell tumors	Dieckmann 2019^a^ [5]	91	44	26; 55	32	15; 50	45.7%	3%
All benign testicular tumors	Hamburg 2022^b^ [26]	79	41	32; 50	10	6; 15	NA	NA
Testicular seminoma	Hamburg 2022^b^ [26]	365	40	33; 48	30	19; 46	53.5%^c^	Isolated cases
Testicular nonseminoma	Hamburg 2022^b^ [26]	179	31	26; 37	35	20; 49	45%^c^	Isolated cases
Other malignant testicular tumors	Hamburg 2022^b^ [26]	18	72.5	68; 78	53	49; 56	NA	0%

*IQR* interquartile range, *TSS* testis-sparing surgery

^a^These publications represent large surveys of the literature

^b^Data of the authors’ institution, detailed in [26]

^c^Data from Dieckmann *et al*. 2018 [27]

It becomes clear that MGST does well to compare with other benign tumors, such as Leydig cell tumors or Sertoli cell tumors, regarding median age, tumor size, and laterality. Also, germ cell tumors, seminomas, and nonseminomas are likewise not much different from MGST with respect to patient age. However, testicular GCTs apparently present with somewhat larger tumor sizes of 30–35 mm than MGSTs (median 20 mm). The rather small size of MGSTs may relate to the very slow growth rate as documented by the KI-67 index of < 1% opposed to KI-67 proliferation rates of 80–95% in GCTs.

Only the group of other malignant testicular neoplasms, such as malignant testicular lymphoma or secondary neoplasms (metastases), comprise markedly larger tumor sizes and older patient ages.

Histological diagnosis of MGST firstly relies on the presence of spindle-shaped cells presenting in a microfibrillar pattern. Secondly, immunohistological workup is needed to detect expression of S100 protein, smooth muscle actin (SMA), and desmin. The latter represents a specific intermediate filament protein that is essential for the structural integrity and function of muscle cells [30]. This immunohistochemical marker provided the final evidence for the myogenic differentiation of the mesenchymal cells that are present in this neoplasm. In contrast to most other gonadal stromal tumors [8], inhibin and calretinin are not necessarily expressed in MGST.

Preoperative diagnosis of MSGST is hardly possible because no specific symptoms prevail. Moreover, ultrasonographic findings are not specific for MGST. However, as shown in the present patient and several previously published cases, the testicular mass in MGST is usually roundish with clear margins. This finding is usually compatible with a benign tumor, particularly if the mass is small [31, 32]. TSS guided by frozen section examination can safely be performed in MGST, although the final diagnosis of this rare neoplasm will certainly be established only upon final immunohistochemical examination. In benign tumors, conservative surgery is principally feasible because no precursor cells of GCT (germ cell neoplasia *in situ*) are present in these testes, and thus, local recurrences are extremely rare. Testis-sparing surgery is particularly beneficial because testosterone deficiencies can be prevented in the later course. Therefore, this type of surgery is advocated by contemporary guidelines in all benign testicular neoplasms, unless the mass is multifocal or larger than 2 cm [33, 34]. In very small masses (< 5 mm), even surveillance without upfront surgery may be considered [35]. In view of the present knowledge about MGST, there is only little doubt about the benign nature of this neoplasm, and thus, a conservative surgical procedure is clearly justified.

Because of the rarity of benign testicular tumors, contemporary guidelines do not give clear recommendations regarding follow-up. Based on the cumulative experience regarding the clinical management of benign testicular tumors, an annual follow-up examination, including scrotal sonography, appears reasonable [33, 36].

## Conclusion

Myoid gonadal stromal tumors represent an emerging new entity of benign testicular gonadal stromal tumors. Immunohistological hallmarks are the expression of desmin, smooth muscle actin, and S100 protein. The clinical characteristics are not much different from those of other benign testicular neoplasms. Testis-sparing surgery appears to be the treatment of choice.

## Data Availability

Data sharing is not applicable to this article as no datasets were generated or analysed during the current study. All clinical data presented in the manuscript are available from the corresponding author upon reasonable request.

## Abbreviations

MGST: Myoid gonadal stromal tumor
GCT: Germ cell tumor
IU: International units
IQR: Inter quartile range
NOS: Not otherwise specified
RL: Reference limit
SMA: Small muscle actin
TSS: Testis-sparing surgery
WHO: World Health Organization
